# Analytical Validation of an LC-MS/MS Method for Simultaneous Quantification of Multiple Immunosuppressants in Microvolume Whole Blood

**DOI:** 10.3390/ijms26136358

**Published:** 2025-07-01

**Authors:** Kenichi Aizawa, Natsuka Kimura, Takahiro Goda, Sho Nishida, Yasunaru Sakuma, Daiki Iwami, Ryozo Nagai

**Affiliations:** 1 Division of Translational Research, Clinical Research Center, Jichi Medical University Hospital, Shimotsuke 329-0498, Tochigi, Japan; 2 Clinical Pharmacology Center, Jichi Medical University Hospital, Shimotsuke 329-0498, Tochigi, Japan; 3 Solutions COE, Analytical & Measuring Instruments Division, Shimadzu Corporation, Kawasaki 210-0821, Kanagawa, Japan; 4 Division of Renal Surgery and Transplantation, Department of Urology, Jichi Medical University, Shimotsuke 329-0498, Tochigi, Japan; 5 Division of Gastroenterological, General and Transplant Surgery, Department of Surgery, Jichi Medical University, Shimotsuke 329-0498, Tochigi, Japan; 6 Jichi Medical University, Shimotsuke 329-0498, Tochigi, Japan

**Keywords:** LC-MS/MS immunosuppressants, microvolume whole blood, hematocrit correction, simultaneous analysis

## Abstract

Immunosuppressants are essential for preventing allograft rejection; however, they require therapeutic drug monitoring to maintain efficacy and to prevent severe complications such as opportunistic infections. Calcineurin inhibitors (CIs) are primarily distributed in red blood cells, whereas mycophenolic acid (MPA) and its metabolites are found in plasma. These differences necessitate separate analyses for each drug, increasing laboratory workload, analytical complexity, and patient burden. We developed a liquid chromatography–tandem mass spectrometry method for simultaneous quantification of CIs such as tacrolimus (Tac), everolimus (Eve), sirolimus (Sir), cyclosporine A (CycA) and MPA in 2.8-µL whole-blood samples, with a hematocrit-based correction to estimate plasma-equivalent MPA concentrations. Performance of this method was assessed by comparison with conventional immunoassay results using linear regression and Bland–Altman analyses, demonstrating excellent agreement, with strong linearity (R^2^ > 0.995) at <2 to 35 ng/mL for three CIs, 26.0 to 1866 ng/mL for CycA, and 0.1 to 50 μg/mL for MPA. Furthermore, MPA and tacrolimus concentrations closely aligned with routine clinical results (R^2^ > 0.900), indicating high accuracy and reproducibility. This new approach may be particularly beneficial for hospitalized patients with limited venous access, pediatric populations, and in remote care settings where frequent blood sampling is challenging because of simultaneous quantification and fewer sample volume requirements.

## 1. Introduction

Immunosuppressive agents are essential for preventing allograft rejection in transplant recipients and for managing autoimmune diseases. However, overdosing can lead to severe complications, including opportunistic infections, underscoring the need for precise dose control through therapeutic drug monitoring (TDM). Several analytical techniques, such as high-performance liquid chromatography (HPLC), liquid chromatography–mass spectrometry (LC-MS), and liquid chromatography–tandem mass spectrometry (LC-MS/MS) are widely used for immunosuppressant quantification [1,2,3,4,5]. In addition, ultra-high-performance liquid chromatography (UHPLC)-LC-MS/MS techniques with high-throughput capabilities and automated dual-channel systems have also been used for efficient immunosuppressant analysis [6,7,8,9]. Solid-phase versus liquid-phase extraction and dried blood spot (DBS) technology for microsampling have also been extensively studied [10,11,12,13,14,15,16].

In this study, we focused on the five most commonly used immunosuppressive agents in post-transplant pharmacotherapy: the CIs such as Tac and CycA, the mTOR inhibitors Eve and Sir, and the antimetabolite MPA as well as MPA’s glucuronide metabolite (MPAG). These agents are widely adopted in clinical practice for TDM, and precise quantification is essential for individualized immunosuppressive therapy and long-term graft survival. Calcineurin inhibitors, including tacrolimus, everolimus, sirolimus, and cyclosporin A, are major immunosuppressants, commonly co-administered with mycophenolic acid, a purine synthesis inhibitor, to enhance immunosuppressive efficacy during early-stage post-transplantation care. However, long-term use of CIs, particularly Tac, which is widely used in kidney transplantation immunosuppressive regimens, leads to various adverse effects, including organ toxicity, highlighting the need for reliable monitoring of alternative or complementary therapeutants [17,18]. Furthermore, unlike CIs, which are primarily distributed in red blood cells, MPA predominantly binds to plasma proteins, necessitating plasma-based quantification [3,7,8,19]. Moreover, the stability of MPA and its metabolites in plasma is well-documented [20]. Therefore, MPA and its metabolites, such as mycophenolic acid β-D-glucuronide, require plasma-based analysis, complicating unified TDM workflows. This distribution difference requires separate pretreatment procedures and analyses for each drug, making simultaneous quantification of all drugs challenging.

Furthermore, advances in microsampling technologies, such as DBS and volumetric absorptive microsampling, have enabled less invasive blood sample collection [15,21,22]. These innovations enable accurate quantification from microvolumes (several µL) of blood, thereby reducing the burden on patients and medical personnel. Devices integrating hematocrit-independent quantification [23,24,25] and plasma separation mechanisms [26,27] have further expanded the potential of microsampling in clinical settings.

In the present study, we developed and validated an LC-MS/MS-based method for simultaneously analyzing major immunosuppressants, suitable for integration with various microsampling devices, and using only 2.8 μL of blood. By unifying pretreatment and analytical workflows for plasma and whole blood analytes, this method can streamline TDM workflows, reduce patient burden, and support precision medicine, particularly in telemedicine and for hospitalized patients with limited venous access. In addition, the purpose of this study is method development and evaluation of the principle at an early stage, for research use only (RUO); it is not intended for clinical or diagnostic research.

## 2. Results and Discussion

### 2.1. Comparison of MPA Concentration in Whole Blood and Plasma

**Figure 1:**
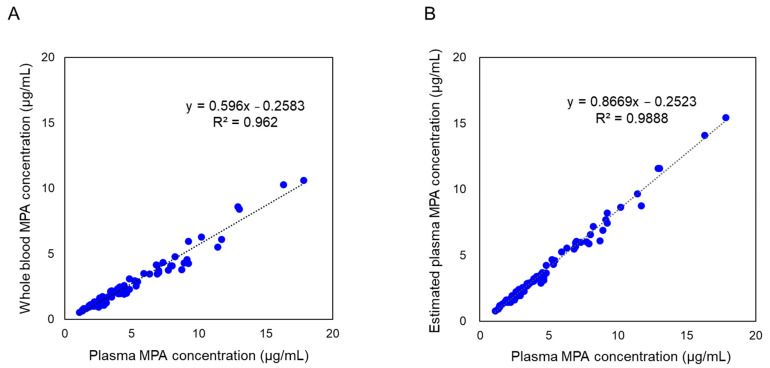
Comparison of mycophenolic acid (MPA) concentrations in whole blood and plasma. (**A**) MPA concentrations in whole-blood samples before applying the hematocrit correction, compared with plasma MPA concentrations obtained through clinical laboratory testing. (**B**) Estimated plasma-equivalent concentrations of MPA, derived using the hematocrit correction formula (Equation (1): Cp = Cwb × 100/(100 − Ht)), compared with plasma MPA concentrations obtained through clinical laboratory testing. n = 72. MPA concentrations in whole blood and estimated plasma were quantified using the developed LC-MS/MS method, whereas plasma MPA concentrations were determined using a separate LC-MS/MS method employed in clinical laboratory testing.

A total of 72 whole-blood samples from 20 patients who underwent renal transplantation were analyzed for estimation of MPA concentrations using a plasma reagent kit. Before hematocrit correction, a correlation between whole-blood and plasma concentrations was observed. However, values differed systematically owing to inherent differences in measurement matrices. The plasma concentration of MPA was lower in whole blood than in plasma (Figure 1A). After applying hematocrit correction, regression analysis yielded the equation y **=** 0.8669x **−** 0.2523, demonstrating strong agreement between estimated plasma concentrations and reference clinical laboratory values (Figure 1B)**.** The coefficient of determination (R^2^ = 0.9888) confirmed a high degree of correlation, demonstrating the robustness of this hematocrit correction method for estimating plasma-equivalent MPA concentrations from whole blood samples.

### 2.2. Evaluation of Simultaneous Analysis of Immunosuppressants

**Figure 2:**
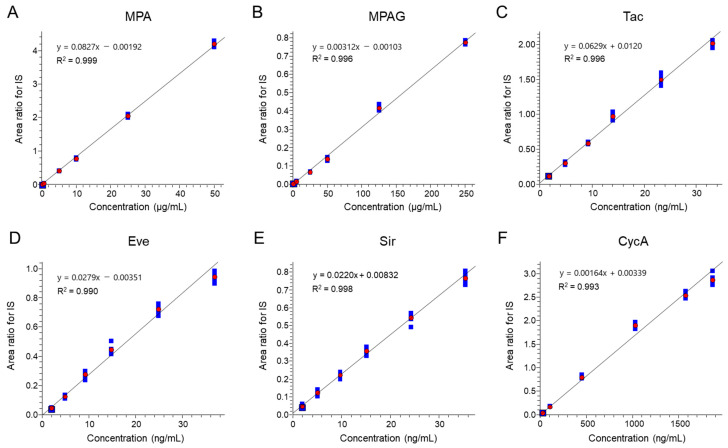
Calibration curves of individual targets. Standard calibration curves of (**A**) MPA, (**B**) MPAG, (**C**) Tac, (**D**) Eve, (**E**) Sir, and (**F**) CycA for the analytical method reported here. Each calibration curve was obtained with the standard concentration in the calibrator that appeared in accordance with the protocol. Values of y- and x-axes were calculated using the internal standard (IS) method with an IS for each immunosuppressant. Red circles represent the average area ratio for IS at each level, and blue squares represent the individual area ratio for IS at each level (n = 6).

**Figure 3:**
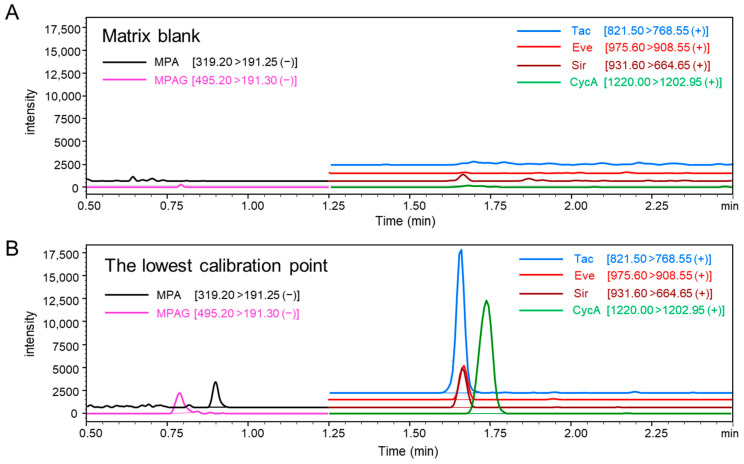
Representative chromatograms of each immunosuppressant. (**A**) Chromatograms of the blank whole blood matrix. (**B**) Chromatograms of analytes at the lowest calibration level.

Simultaneous quantification of MPA, MPAG, and CIs (Tac, Eve, Sir, and CycA) was achieved using ESI-negative mode for MPA/MPAG and ESI-positive mode for Tac, Eve, Sir, and CycA. Detector responses to MPA and MPAG analyzed in ESI-negative mode were comparable to those of CIs analyzed in ESI-positive mode with identical injection volumes. Calibration curves demonstrated high linearity, with R^2^ values of 0.999 for MPA, 0.996 for MPAG, and between 0.990 and 0.998 for the calcineurin inhibitors (Figure 2). Representative chromatograms (Figure 3) show well-defined peaks for all analytes, with MPA and MPAG exhibiting intensities comparable to those of CIs.

Well-defined peaks are shown for MPA (0.1 µg/mL), MPAG (1.0 µg/mL), Tac (1.73 ng/mL), Eve (2.04 ng/mL), Sir (1.93 ng/mL), and CycA (26.07 ng/mL). MPA and MPAG were analyzed in electrospray ionization (ESI)-negative mode, whereas Tac, Eve, Sir, and CycA were analyzed in ESI-positive mode. Relative intensity levels of MPA and MPAG were comparable to those of other immunosuppressants among chromatograms.

Quality control (QC) assessments, with six replicates at three concentrations for MPA and MPAG and four concentrations for calcineurin inhibitors, showed relative standard deviations (RSDs) consistently below 10%. Accuracy was within ±15% for all analytes, further validating the robustness of the method (Table 1). In addition, carryover was also assessed for all analytes. As a result, carryover of five analytes except for CycA was less than 20% to the lowest calibration point (L1) as shown in Table S1. However, CycA showed 28.0% as carryover [28].

### 2.3. Cross-Validation at the Two Sites

The assay was evaluated at two independent laboratories (Jichi Medical University and Shimadzu Corporation) for validation. Results demonstrated accuracy in recovery from 80% to 120% of expected values, meeting bioanalytical method validation guidelines (Table S2). The method exhibited strong robustness, as the majority of samples fell within acceptable limits.

### 2.4. Comparative Analysis—Simultaneous Assessment of MPA and Tac with Microvolume Sampling and Clinical Laboratory Testing

MPA and Tac are either prescribed individually or in combination for most patients; therefore, we selected these two analytes for verification. This novel LC-MS/MS method using microvolume sampling (2.8 μL) for MPA and Tac demonstrated a high correlation with clinical laboratory testing.

**Figure 4:**
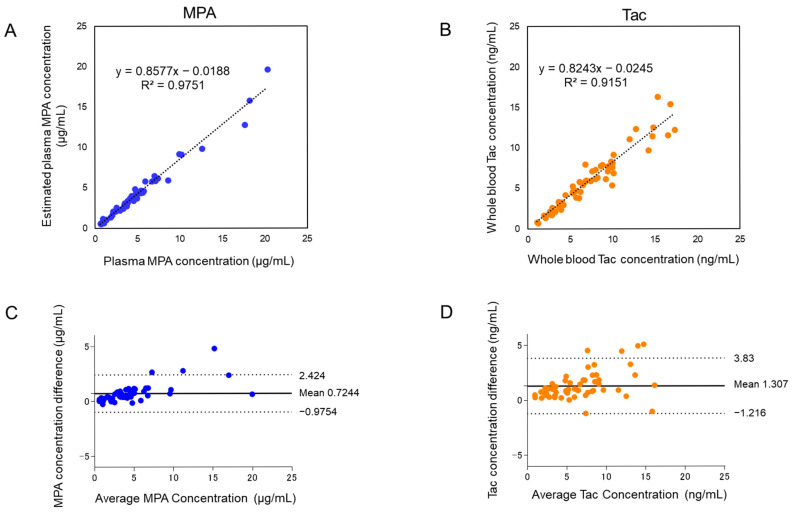
Comparison of MPA and Tac results between the developed method and clinical laboratory testing. (**A**) Linear regression analysis of MPA concentrations, comparing the developed method with hematocrit correction and clinical laboratory testing (LC-MS/MS). (**B**) Linear regression analysis of Tac concentrations comparing the developed method and clinical laboratory testing (immunoassay). (**C**) Bland–Altman difference plot showing agreement of MPA concentrations between the two methods. (**D**) Bland–Altman difference plot showing agreement of Tac concentrations between the two methods. A total of 58 samples from 15 patients were analyzed. Both the developed microvolume LC-MS/MS assay and the LC-MS/MS method employed in clinical laboratory testing demonstrated strong correlations and minimal biases for MPA and Tac concentrations. Bland–Altman analysis, illustrated in (**C**,**D**), confirmed the comparability of the two methods, with the majority of data points falling within acceptable limits of agreement.

Linear regression analysis for MPA (R^2^ = 0.9751) and Tac (R^2^ = 0.9151) confirmed the reliability of the microvolume assay (Figure 4A,B). Estimation of hematocrit-corrected plasma-equivalent concentrations and Bland–Altman analysis confirmed minimal bias and strong agreement between the two methods, with most data points falling within acceptable limits of agreement (Figure 4C,D).

In this study, comparative analysis of the developed LC-MS/MS method with conventional methods was assessed for 58 samples. To verify the reliability of the immunosuppressant concentrations, a portion of sample (22 samples) was reanalyzed for MPA and Tac (Table S3). More than 70% of repeating samples had percent differences within ±20% for MPA and Tac, respectively [28]. The developed method proved to have a high reliability.

### 2.5. Discussion

Tac has long been the cornerstone of immunosuppressive therapy, particularly for transplant recipients. However, its long-term use is associated with significant adverse effects, including nephrotoxicity and organ damage [17,18]. Our study further highlights renal toxicity linked to carnitine deficiency in renal tissue, as observed in metabolic studies using mouse models [29,30]. These findings underscore the importance of precise TDM to minimize adverse effects and to optimize long-term immunosuppressive regimens.

Simultaneously quantifying Tac and MPA, which are often prescribed together for patients who underwent transplantation, presents unique analytical challenges. MPA is primarily quantified in plasma due to its strong binding to plasma proteins, while Tac and other CIs are measured in whole blood. Substantial differences in concentration ranges and detector responses to these analytes have previously necessitated separate analytical workflows.

In this study, we developed and evaluated a novel LC-MS/MS method that enables simultaneous quantification of Tac, MPA, MPAG, and other major CIs, such as Eve, Sir, CycA, and MPAG. A key innovation of our method is the use of ESI-negative mode for MPA and MPAG and ESI-positive mode for Tac, Eve, Sir, and CycA. For simultaneous analysis of immunosuppressants with different effective concentration ranges in whole blood, the combination of negative and positive modes was helpful to control the signal intensity in a single analysis. Furthermore, without the need for separate preparations for each sample type, such as plasma or whole blood, according to the target immunosuppressant, hematocrit-based correction allowed for estimation of plasma-equivalent MPA concentrations directly from whole blood, ensuring reliable and consistent results.

Our method demonstrated robust performance for simultaneous quantification across analytes, validated by QC samples and actual patient samples. Despite slightly reduced sensitivity in ESI-negative mode for MPA and MPAG, peak intensities remained sufficient for accurate quantification without significant background interference (Figure 3). QC analysis showed RSD values consistently below 15%, with accuracy within the acceptable range of 80–120% of theoretical values (Table 1). These findings confirm the method’s reliability for routine clinical use.

Cross-validation at two independent laboratories demonstrated the robustness of the method, as results conformed to bioanalytical validation guidelines, with assay variability within ±20% for at least two-thirds of the samples [31]. This consistency across sites confirms the developed method’s adaptability for clinical applications.

Analytical efficiency was further demonstrated by optimizing the cycle time to approximately 3 min, enabling simultaneous analysis of Tac, Eve, Sir, CycA, MPA, and MPAG. This represents a significant improvement over previously reported UHPLC methods, which typically require 2.5 to 3.5 min per analyte [7,8,16]. The ability to analyze multiple immunosuppressants in a single injection within a 3 min cycle enhances workflow efficiency, particularly in high-throughput clinical and research settings.

Agreement between our LC-MS/MS method and conventional techniques was further validated through correlation analyses and Bland–Altman plots (Figure 4A–D). While immunoassay results for Tac showed systematically higher concentrations due to known cross-reactivity, our LC-MS/MS approach offered superior selectivity through multiple reaction monitoring (MRM), significantly reducing interference. Bland–Altman analysis indicated that most concentration differences fell within the 95% limits of agreement, with outliers observed in only 6.0% and 7.0% of cases for MPA and Tac, respectively, confirming the method’s robustness and clinical reliability.

A major advantage of our method is its adaptability to microsampling. By reducing the sample volume to only 2.8 μL of whole blood—less than one-tenth of the volume required by standard protocols—our method is compatible with microsampling devices, making it ideal for patients with limited blood volume, such as pediatric or critically ill patients [17,24,25]. This minimal sample requirement, combined with high-throughput capabilities, also enhances the method’s suitability for telemedicine applications, where remote monitoring of immunosuppressant levels can significantly improve patient care, particularly for individuals with limited access to specialized healthcare.

The capacity to simultaneously quantify multiple immunosuppressants, even when measured in different matrices such as plasma or whole blood, constitutes a substantial advance in TDM. This method not only streamlines analytical workflows but also facilitates evaluation of adverse effects associated with long-term immunosuppressive therapy. By integrating microvolume sampling with efficient LC-MS/MS analysis, the developed approach reduces time and effort required for sample collection and processing, enhancing its clinical utility. In addition, its potential application in telemedicine offers a promising avenue for expanding access to personalized and timely therapeutic monitoring. The capacity to process microvolume samples from remote locations could reduce the burden on patients and healthcare systems, making it invaluable in this era of digital healthcare.

In conclusion, this study presents a validated LC-MS/MS method for simultaneous analysis of major immunosuppressants in whole blood. By addressing key analytical challenges, our method facilitates precision TDM, optimizing therapy for transplant recipients and other patients requiring long-term immunosuppressive treatment. Although this study was for RUO purposes, future work will apply this method to additional therapeutic agents and will further evaluate its performance in multi-sample clinical workflows.

## 3. Materials and Methods

### 3.1. Chemical and Reagents

DOSIMMUNE^TM^ reagent kits (Shimadzu Corporation, Kyoto, Japan) were used for quantification of Tac, Eve, Sir, and CycA in whole blood. DOSIMYCO^TM^ reagent kits (Shimadzu Corporation) were used for analysis of MPA and MPAG in plasma. Both kits are for RUO purposes and include essential components, such as reversed phase chromatographic columns, methanol-based mobile phases, isotope-labeled internal standards (IS), a calibrator set, quality control (QC) materials, and sample extraction reagents. Batch numbers for individual reagents in DOSIMMUNE^TM^ reagent kits were IMCAB22001, IMCAB20009, and IMCAB23003 for the calibration set; IMREC22004 for internal standards; and IMQCB22001 for the QC set, respectively. The batch number of DOSIMYCO^TM^ internal standard was MYSIL21001. These ISs were labeled with a combination of ^13^C and ^2^H and [^13^C, ^2^H_4_]-Tac, [^13^C_2_, ^2^H_4_]-Eve, [^13^C, ^2^H_3_]-Sir, and [^2^H_12_]-CycA were used for each CI. The other ISs were [^13^C, ^2^H_3_]-MPA and [^13^C, ^2^H_3_]-MPAG. Independent standards for MPA and MPAG of more than 95% purity were procured from Alsachim (Illkirch-Graffenstaden, Bas-Rhin, France). Chemical structures of the six target compounds are illustrated in Figure S1. Normal human-control whole-blood samples (with EDTA-2K added) were purchased from BizCom Japan, Inc. (Tokyo, Japan) for validation purposes. LC-MS-grade methanol was obtained from FUJIFILM Wako Pure Chemical Corporation (Osaka, Japan) and Milli-Q-grade water was prepared using the Milli-Q IQ 7015 water purification system (Merck, Tokyo, Japan).

### 3.2. Hematocrit Correction for MPA Levels in Whole-Blood Samples

Quantitative measurements of MPA in whole blood were adjusted for hematocrit values, which were determined prior to analysis. Plasma-equivalent MPA concentrations were estimated using the following conversion formula:

eCp = Cwb × 100/(100 − Ht)(1)

where

**eCp** represents the estimated concentration in plasma;
**Cwb** is the measured concentration in whole blood;
**Ht** denotes the hematocrit value (percentage).

This correction enables estimation of plasma-equivalent concentrations directly from whole-blood samples, allowing for more accurate comparisons with conventional plasma-based assays.

### 3.3. Instrumentation and Analytical Conditions for Simultaneous LC-MS/MS Analysis of Immunosuppressants

Simultaneous analysis of immunosuppressants was conducted using an LCMS-8050 triple quadrupole mass spectrometer (Shimadzu Corporation, Kyoto, Japan) fitted with LC-30AD pumps and a SIL-30ACMP autosampler. Mobile phases A and B, trapping and analytical columns, and other reagents were included in the DOSIMMUNE^TM^ kit. Mobile phase compositions are proprietary to the supplier and are not disclosed. Analytical conditions were optimized for concurrent quantification of immunosuppressants.

The gradient elution program began with 60% Buffer B, held for 1 min, followed by a linear increase to 100% over 1.25 min. The concentration of Buffer B was maintained at 100% for an additional 1 min using a binary gradient system. The flow rate was 0.8 mL/min, and the column oven was maintained at 65 °C. The sample loading column was switched to the analytical flow path at 0.25 min and reverted to the waste position at 1.5 min, with a loading flow rate of 2 mL/min. Mass spectrometric parameters were as follows: nebulizer gas flow at 3 L/min, heating gas flow at 10 L/min, drying gas flow at 10 L/min, interface temperature at 200 °C, and de-solvation line and heat block temperatures at 150 °C and 200 °C, respectively.

MPA, its isotope-labeled internal standard (MPA-IS), MPAG, and MPAG-IS were quantified using MRM transitions of 319.20 > 191.25 (MPA), 323.20 > 191.25 (MPA-IS), 495.20 > 191.30 (MPAG), and 499.20 > 191.20 (MPAG-IS), respectively, in negative-ESI mode. Negative-ion detection confirmed equivalent response intensities for MPA, MPA-IS, MPAG, and MPAG-IS compared to Tac, Eve, Sir, and CycA. Additional MRM transitions, such as 319.20 > 275.30 (MPA) and 495.20 > 113.20 (MPAG), were included for qualification monitoring. For Tac, Eve, Sir, and CycA, MRM transitions were optimized according to definitions provided in the reagent kit for immunosuppressants in whole blood. Detailed MRM parameters for all analytes are summarized in Table S4. Following extraction, 20 μL of sample was injected into the analytical system using a trap injection mechanism. A schematic diagram of the system configuration is shown in Figure S2.

### 3.4. Preparation of Calibration Standards and QC Samples

Calibration standards for simultaneous analysis were prepared by supplementing the calibrators provided in the reagent kit (which included Tac, Eve, Sir, and CycA) with their respective isotope-labeled IS, and by adding independent standards for MPA and MPAG. Standard solutions for MPA and MPAG were prepared at six concentrations (L1 to L6).

To prepare these standards, dried MPA and MPAG were reconstituted in 2.8 μL of the L1 solution from the reagent kit, along with IS solutions from reagent kits for whole blood and plasma (Figure S3). The mixture was vortexed, centrifuged, and diluted to achieve desired concentrations. Calibration levels L2 through L6 were prepared in the same manner. Final concentrations of other immunosuppressants were defined according to the manufacturer’s specifications, as outlined in the reagent kit for whole blood. All calibration details are presented in Table S5.

QC samples for MPA (2, 20, 40 μg/mL) and MPAG (10, 100, 200 μg/mL) were prepared using human-control whole-blood samples purchased from an external vendor, following the same protocol used for calibrators. Certified QC samples included in the reagent kit for whole blood were used as QC samples of Tac, Eve, Sir, and CycA. Specified concentrations of QC samples are 3.3, 7.6, 12.7, and 18.6 ng/mL for Tac; 3.9, 9.1, 12.3, and 20.8 ng/mL for Eve; 3.8, 8.4, 11.9, and 20.8 ng/mL for Sir; and 42.5, 157.2, 775.5, and 1357.6 ng/mL for CycA, respectively. Six QC samples were prepared for each analyte to validate accuracy and reproducibility of the assay [28].

### 3.5. Cross-Validation of the Developed Method at Different Sites

Cross-validation was performed independently in the laboratory at Jichi Medical University and in the laboratory of Shimadzu Corporation. Eight whole-blood working-test samples were prepared with eight concentrations of Eve, Tac, MPA, and MPAG and added to purchased control whole blood (which had not been treated with immunosuppressants). Added concentrations of Eve and Tac were 2.4, 3.0, 6.0, 7.5, 12.0, 15.0, 24.0, and 30.0 ng/mL; those of MPA were 0.24, 0.30, 6.0, 7.5, 12.0, 15.0, 24.0, and 30 µg/mL; and MPAG were 1.2, 1.5, 30.0, 37.50, 60.0, 75.0, 120.0, and 150.0 µg/mL. All prepared samples were dispensed and frozen for analysis at each facility. Samples were then shipped and stored at −80 °C until sample preparation.

A different operator at each site analyzed samples to assess the validity of the developed method. A 2.8-µL sample of whole blood was collected from each test sample, and sample preparation was carried out following the same protocol described for Calibration Standards and QC Samples at each site. Each test sample was analyzed six times, and the average recovery ratio was calculated for each. Results were evaluated according to guidelines for bioanalytical cross-validation [31].

### 3.6. Comparison of Clinical Laboratory Testing with Microvolume Assays of Whole Blood

Blood samples were collected from superficial veins using a tourniquet at four time points (08:00, 09:00, 11:00, and 14:00) daily from 15 hospitalized patients, yielding a total of 58 samples. This study was conducted in accordance with the Declaration of Helsinki and approved by the Bioethics Committee for Clinical Research at Jichi Medical University Hospital (approval code: CU20-064; approval date: 10 February 2021). Written informed consent was obtained from all participants involved in this study. Each sample was divided into whole blood and plasma fractions, and routine quantification was performed following clinical practice guidelines of the hospital laboratory. Whole blood samples intended for microvolume analysis were stored at −80 °C until processing.

For the microvolume assay, IS specific to each analyte was added to whole blood samples. Following centrifugation, 20 μL of supernatant were injected into the LC-MS/MS analytical system for quantification. Results of the microvolume whole-blood assay were compared with those from routine clinical laboratory testing performed on plasma or whole blood, depending on the immunosuppressant.

To enable direct comparison with plasma-based clinical laboratory test results, plasma-equivalent MPA concentrations were estimated using the hematocrit correction formula (Equation (1)) described earlier. Method comparison and statistical analyses were conducted using GraphPad PRISM 7 for Windows (version 7.04). Agreement between the conventional immunoassay-based clinical laboratory methods and the developed LC-MS/MS microvolume assay was evaluated using Bland–Altman regression analysis.

### 3.7. Measurement Method for MPA and Tac in Clinical Laboratory Testing

To measure MPA concentrations in whole blood and to compare the developed LC-MS/MS method with conventional clinical laboratory testing, whole-blood samples were collected from patients at Jichi Medical University Hospital in accordance with local medical ethical regulations. Samples were drawn four times (08:00, 09:00, 11:00, and 14:00) daily from 20 patients, yielding a total of 72 samples.

Blood samples were fractionated into whole blood and plasma. Plasma MPA concentrations were quantified using LC-MS/MS at an external testing facility, following the facility’s clinical practice guidelines. Whole-blood samples intended for MPA analysis were stored at −80 °C until processing, and sample preparation was performed according to the analytical protocol provided in the reagent kit for plasma.

Tac concentrations in whole blood were determined using an electrochemiluminescence immunoassay on a Cobas 8000 analyzer (Roche Diagnostics) in the hospital’s clinical laboratory. Plasma MPA concentrations obtained from the external facility were used as a reference for comparison. All procedures adhered to clinical practice guidelines of the hospital and external testing laboratory to ensure consistency and reliability.

## Figures and Tables

**Table 1:** Summary of QC results for each immunosuppressant.

QC Sample		Immunosuppressant		Level							
				1		2		3		4	
				Conc. (avg.)	Accuracy (%)	Conc. (avg.)	Accuracy (%)	Conc. (avg.)	Accuracy (%)	Conc. (avg.)	Accuracy (%)
					%RSD		%RSD		%RSD		%RSD
Whole blood	1	MPA	(µg/mL)	2.22	111	19.5	98	40.1	100	-	-
					2.46		1.92		1.21		-
	2			2.07	104	18.96	95	43.41	109	-	-
					2.93		1.94		1.27		-
	3			2.10	105	19.56	98	40.73	102	-	-
					0.92		1.32		2.49		-
	4			1.94	97	20.83	104	43.72	109	-	-
					3.04		2.19		2.46		-
	5			1.98	99	20.03	100	40.17	100	-	-
					2.76		0.86		0.51		-
	6			2.08	104	20.46	102	39.43	99	-	-
					3.68		1.32		2.55		-
	1	MPAG	(µg/mL)	10.1	101	97.34	97	199.6	100	-	-
					2.63		2.13		2.3		-
	2			10.9	109	93.28	93	180.9	90	-	-
					2.56		3.30		2.04		-
	3			9.72	97	98.71	99	203.0	102	-	-
					8.56		3.10		1.03		-
	4			9.51	95	97.02	97	214.6	107	-	-
					9.32		5.92		1.08		-
	5			9.49	95	103.0	103	206.6	103	-	-
					5.23		1.47		1.36		-
	6			10.3	103	92.72	93	195.8	98	-	-
					5.30		3.31		1.68		-
Control (reagent kit for whole blood)		Tac	(ng/mL)	3.17	94	7.69	100	12.07	95	18.83	101
					6.31		5.65		3.05		3.11
		Eve		3.83	98	9.80	107	14.01	113	21.64	104
					5.57		7.86		4.06		7.69
		Sir		3.99	104	9.48	112	12.09	101	19.38	93
					5.34		3.07		4.87		6.75
		CycA		40.8	96	169.8	108	822.1	106	1412	104
					4.69		7.10		3.42		1.44

QC results include relative standard deviations (RSDs) and accuracy values for MPA, MPAG, Tac, Eve, Sir, and CycA. These results demonstrate the reproducibility and reliability of the developed method at different concentrations.

## Data Availability

Datasets generated and/or analyzed during this study are available from the corresponding author upon request.

## Supplementary Materials

The following supporting information can be downloaded at: https://www.mdpi.com/article/10.3390/ijms26136358/s1.

## Abbreviations

LC-MS/MS	liquid chromatography–tandem mass spectrometry
UHPLC	ultra-high-performance liquid chromatography
TDM	therapeutic drug monitoring
MPA	mycophenolic acid
MPAG	mycophenolic acid β-D-glucuronide
CIs	calcineurin inhibitors
Tac	tacrolimus
Eve	everolimus
Sir	sirolimus
CycA	cyclosporin A
DBS	dried blood spot
MRM	multiple-reaction monitoring

## References

[1] Deters M., Kaever V., Kirchner G.I. (2003). Liquid chromatography/mass spectrometry for therapeutic drug monitoring of immunosuppressants. Anal. Chim. Acta.

[2] Wiwattanawongsa K., Heinzen E.L., Kemp D.C., Dupuis R.E., Smith P.C. (2001). Determination of mycophenolic acid and its phenol glucuronide metabolite in human plasma and urine by high-performance liquid chromatography. J. Chromatogr. B Biomed. Sci. Appl..

[3] Freudenberger K., Hilbig U., Gauglitz G. (2016). Recent advances in therapeutic drug monitoring of immunosuppressive drugs, Trends. Anal. Chem..

[4] Annesley T.M., McKeown D.A., Holt D.W., Mussell C., Champarnaud E., Harter L., Calton L.J., Mason D.S. (2013). Standardization of LC-MS for therapeutic drug monitoring of tacrolimus. Clin. Chem..

[5] McShane A.J., Bunch D.R., Wang S. (2016). Therapeutic drug monitoring of immunosuppressants by liquid chromatography–mass spectrometry. Clin. Chim. Acta.

[6] Gong Z.S., Wu Z.H., Xu S.X., Han W.N., Jiang X.M., Liu H.P., Wei Y.L., Yan W.Y. (2019). A high-throughput LC-MS/MS method for the quantification of four immunosuppressants drugs in whole blood. Clin. Chim. Acta.

[7] Klepacki J., Klawitter J., Bendrick-Peart J., Schniedewind B., Heischmann S., Shokati T., Christians U., Klawitter J. (2012). A high-throughput U-HPLC–MS/MS assay for the quantification of mycophenolic acid and its major metabolites mycophenolic acid glucuronide and mycophenolic acid acyl-glucuronide in human plasma and urine. J. Chromatogr. B Analyt. Technol. Biomed. Life Sci..

[8] Brandhorst G., Streit F., Goetze S., Oellerich M., Heischmann S., Armstrong V.W. (2006). Quantification by liquid chromatography tandem mass spectrometry of mycophenolic acid and its phenol and acyl glucuronide metabolites. Clin. Chem..

[9] Zijp T.R., Hateren K.V., Kuiper H., Jongedijk E.M., Touw D.J. (2023). Ultrahigh throughput dual channel liquid chromatography with tandem mass spectrometry for quantification of four immunosuppressants in whole blood for therapeutic drug monitoring. J. Chromatogr. A.

[10] Roszkowska A., Treder N., Plenis A., Miękus N., Olędzka I., Kowalski P., Bączek T. (2012). Optimization and comparison of two microsampling approaches for LC-MS/MS analysis of a panel of immunosuppressants in blood samples. Sustain. Chem. Pharm..

[11] Deprez S., Stove C.P. (2023). Dried blood microsampling-assisted therapeutic drug monitoring of immunosuppressants: An overview. J. Chromatogr. A.

[12] Deprez S., Stove C.P. (2021). Fully Automated Dried Blood Spot Extraction coupled to Liquid chromatography-tandem mass spectrometry for Therapeutic Drug Monitoring of immunosuppressants. J. Chromatogr. A.

[13] Deprez S., Van Uytfanghe K.V., Stove C.P. (2023). Liquid chromatography-tandem mass spectrometry for therapeutic drug monitoring of immunosuppressants and creatinine from a single dried blood spot using the Capitainer^®^ qDBS device. Anal. Chim. Acta.

[14] Zwart T.C., Gokoel S.R.M., van der Boog P.J.M., de Fijter J.W., Kweekel D.M., Swen J.J., Guchelaar H.J., Moes D.J.A.R. (2018). Therapeutic drug monitoring of tacrolimus and mycophenolic acid in outpatient renal transplant recipients using a volumetric dried blood spot sampling device. Br. J. Clin. Pharmacol..

[15] Kip A.E., Kiers K.C., Rosin H., Schellens J.H.M., Beijnen J.H., Dorlo T.P.C. (2017). Volumetric absorptive microsampling (VAMS) as an alternative to conventional dried blood spots in the quantification of miltefosine in dried blood samples. J. Pharm. Biomed. Anal..

[16] Rosé G., Tafzi N., Balkhi S.E., Rerolle J.P., Debette-Gratien M., Marquet P., Saint-Marcoux F., Monchaud C. (2023). New perspectives for the therapeutic drug monitoring of tacrolimus: Quantification in volumetric DBS based on an automated extraction and LC-MS/MS analysis. J. Chromatogra. B.

[17] Farouk S.S., Rein J.L. (2020). The many faces of calcineurin inhibitor toxicity-what the FK?. Adv. Chronic Kidney Dis..

[18] Wojciechowski D., Wiseman A. (2021). Long-term immunosuppression management: Opportunities and uncertainties. Clin. J. Am. Soc. Nephrol..

[19] Winnicki W., Fichtenbaum A., Mitulovič G., Herkner H., Regele F., Baier M., Zelzer S., Wagner L., Sengoelge G. (2022). Individualization of mycophenolic acid therapy through pharmacogenetic, pharmacokinetic and pharmacodynamic testing. Biomedicines.

[20] de Loor H., Naesens M., Verbeke K., Vanrenterghem Y., Kuypers D.R. (2008). Stability of mycophenolic acid and glucuronide metabolites in human plasma and the impact of deproteinization methodology. Clin. Chim. Acta.

[21] Lei B.U.W., Prow T.W. (2019). A review of microsampling techniques and their social impact. Biomed. Microdevices.

[22] Deprez S., Paniagua-González L., Velghe S., Stove C.P. (2019). Evaluation of the performance and hematocrit independence of the HemaPEN as a volumetric dried blood spot collection device. Anal. Chem..

[23] Kim J.H., Woenker T., Adamec J., Regnier F.E. (2013). Simple, miniaturized blood plasma extraction method. Anal. Chem..

[24] Deprez S., Heughebaert L., Boffel L., Stove C.P. (2023). Application of non-contact hematocrit prediction technologies to overcome hematocrit effects on immunosuppressant quantification from dried blood spots. Talanta.

[25] Spooner N., Denniff P., Michielsen L., De Vries R., Ji Q.C., Arnold M.E., Woods K., Woolf E.J., Xu Y., Boutet V. (2015). A device for dried blood microsampling in quantitative bioanalysis: Overcoming the issues associated with blood hematocrit. Bioanalysis.

[26] Paniagua-González L., Lendoiro E., Otero-Antón E., López-Rivadulla M., De-Castro-Ríos A., Cruz A. (2022). Comparison of conventional dried blood spots and volumetric absorptive microsampling for tacrolimus and mycophenolic acid determination. J. Pharm. Biomed. Anal..

[27] Ishida H.K., Noritake T., Mano K.Y. (2021). Quantitative and qualitative application of a novel capillary microsampling device, Microsampling Wing^TM^ (MSW), using antiepileptic drugs in rats. J. Pharm. Biomed. Anal..

[28] US Food and Drug Administration (2018). Bioanalytical Method Validation. https://www.fda.gov/files/drugs/published/Bioanalytical-Method-Validation-Guidance-for-Industry.pdf.

[29] Nishida S., Ishima T., Kimura N., Iwami D., Nagai R., Imai Y., Aizawa K. (2024). Metabolomic profiling of mice with tacrolimus-induced nephrotoxicity: Carnitine deficiency in renal tissue. Biomedicines.

[30] Nishida S., Ishima T., Iwami D., Nagai R., Aizawa K. (2025). Whole Blood Metabolomic Profiling of Mice with Tacrolimus-Induced Chronic Nephrotoxicity: NAD+ Depletion with Salvage Pathway Impairment. Antioxidants.

[31] (2013). Pharmaceutical and Medical Device Agency under the Ministry of Health, Labor and Welfare in Japan. *4.3 Cross Validation: Guideline on Bioanalytical Method Validation in Pharmaceutical Development*. https://www.pmda.go.jp/files/000206209.pdf.

