# Supplementary material for: Analytical Validation of an LC-MS/MS Method for Simultaneous Quantification of Multiple Immunosuppressants in Microvolume Whole Blood
Source: Int J Mol Sci. 2025 Jul 1;26(13):6358. doi: 10.3390/ijms26136358 (PMC12249770; doi:10.3390/ijms26136358)
Supplement: Supplementary file 1 [file ijms-26-06358-s001.zip › ijms-3694792-supplementary.pdf]

Supplementary Materials for

Analytical Validation of LC-MS/MS Method for Simultaneous Quantification of Multiple Immunosuppressants in Microvolume Whole Blood

This file includes:

Figs. S1 to S3

Tables S1 to S5

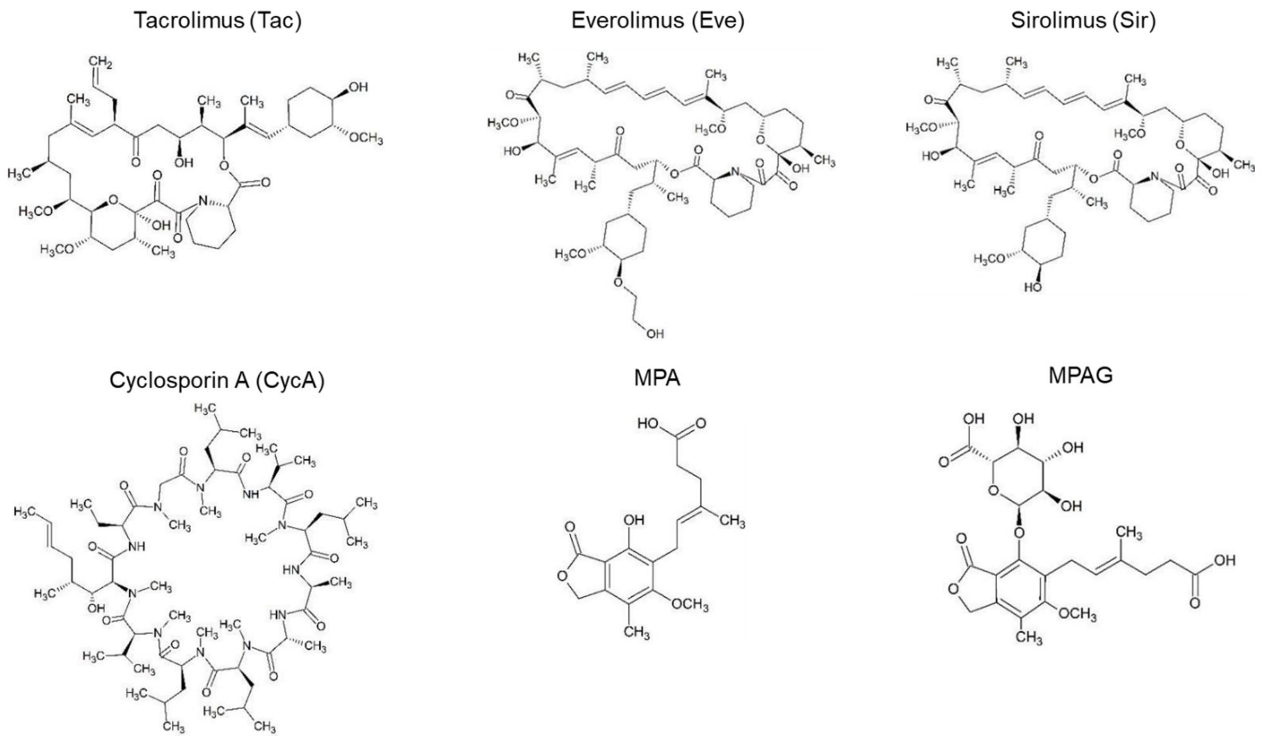

Figure S1. Chemical structures of five immunosuppressants and a metabolite.

Chemical structure of Tacrolimus (Tac), Everolimus (Eve), Sirolimus (Sir), Cyclosporin A (CycA), Mycophenolic acid (MPA), and Mycophenolic acid β-D-glucuronide (MPAG).

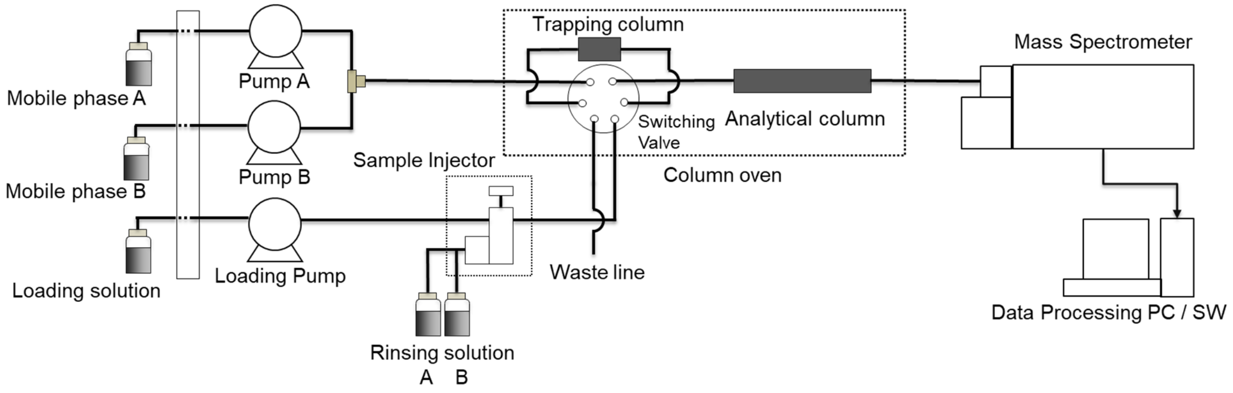

Figure S2. Diagrammatic representation of the LC-MS/MS system.

This system consists of a front-HPLC combined with an MS/MS system. The system has a trapping injection mechanism with a trapping column on which injected samples are concentrated, and desalting is performed. The default sample volume was 20 μL.

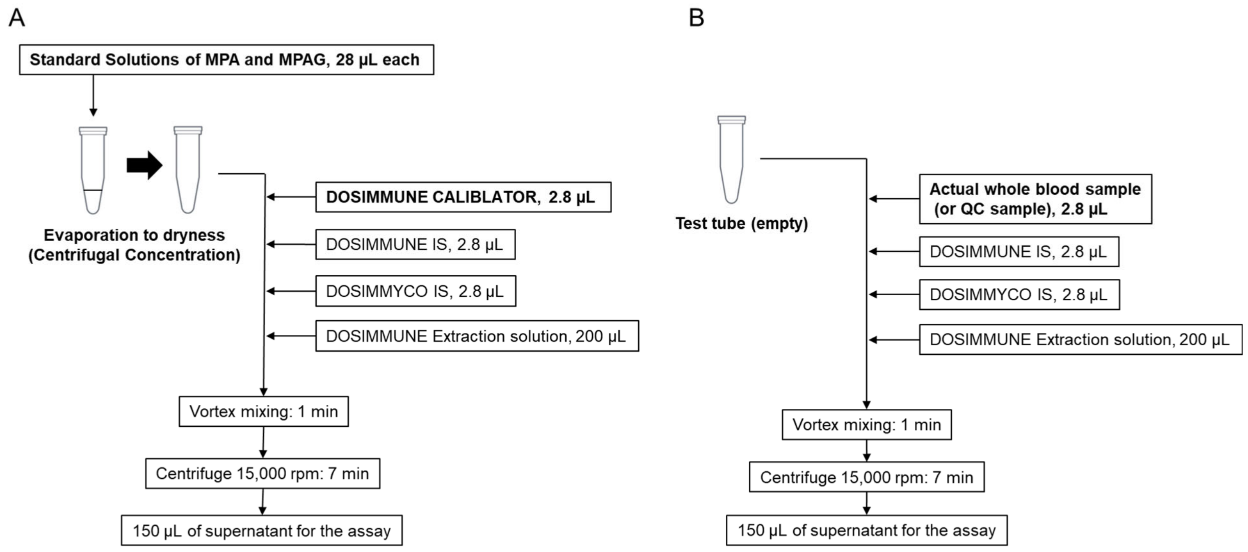

Figure S3. Preparation protocol for the calibrator (A) and sample or QC (B).

For each calibrator, 28 µL of MPA and MPAG methanol solution were added to the test tube followed by evaporation to dryness. Twenty-eight µL were adapted to 2.8 µL of other solutions, and each IS as well as the actual sample volume. This procedure for sample preparation is similar to that for preparation of the calibrator, except for the volume of MPA and MPAG standard and w/ or w/o of dryness.

Table S1. Carryover of each immunosuppressant.

Immunosuppressant	Peak area at the lowest calibrator	Peak area at the highest calibrator	Blank samples following the highest calibrator	Carryover (%)
MPA	3,991	1,981,573	440	11.0
MPAG	3,606	976,826	638	17.7
Eve	6,539	129,331	434	6.6
Sir	7,147	115,558	921	12.9
Tac	29,112	477,636	1,564	5.4
CycA	36,841	2,338,624	10,312	28.0

Peak area of each immunosuppressant means the averaged peak area of the lowest or highest calibration point (L1 or L6). Analysis by LC-MS/MS was repeated six times at each calibration point Peak areas of blank samples shows results of measurement following the highest calibration point (L6). Carryover was assessed by analyzing blank samples after the calibration standard at the ULOQ.

Table S2. Summary of cross-validation for MPA and Tac.

		Level							
		1	2	3	4	5	6	7	8
		Accuracy (%)							
Site 1	MPA (μg/mL)	116	106	111	113	115	116	121*	119
	Tac (ng/mL)	79*	89	78*	88	83	93	97	92
Site 2	MPA (μg/mL)	63*	71*	92	98	97	100	98	97
	Tac (ng/mL)	78*	76*	88	82	86	82	94	91

Averaged accuracy as recovery for each site for MPA and Tac in whole-blood test samples. These results meet guideline criteria in samples more than two-thirds using study samples.

Values that do not meet the criteria are highlighted with an asterisk.

Site 1: Jichi Medical University, Site 2: Shimadzu Corporation.

Table S3. Summary of reanalyzed MPA and Tac

MPA					Tac				
Sample	Initial value (μg/mL)	Repeat value (μg/mL)	Mean value	% difference	Sample	Initial value (ng/mL)	Repeat value (ng/mL)	Mean value	% difference
1	2.45	2.32	2.39	-5.70	1	11.95	12.63	12.29	5.57
2	4.66	5.20	4.93	10.96	2	10.72	15.73	13.22	37.83
3	15.48	13.14	14.31	-16.36	3	17.55	27.88	22.71	45.47
4	3.27	3.30	3.28	0.78	4	17.38	18.98	18.18	8.81
5	0.48	0.48	0.48	0.69	5	4.52	3.51	4.02	-25.02
6	0.74	0.74	0.74	-0.95	6	3.76	4.22	3.99	11.35
7	0.96	1.03	0.99	7.13	7	3.84	4.34	4.09	12.26
8	1.62	1.45	1.54	-11.13	8	4.57	4.84	4.70	5.91
9	7.43	6.61	7.02	-11.70	9	6.84	11.07	8.96	47.19
10	2.13	1.63	1.88	-26.87	10	9.33	10.62	9.98	12.98
11	0.97	0.58	0.78	-50.71	11	9.79	8.78	9.28	-10.89
12	8.91	9.94	9.43	10.98	12	19.11	16.60	17.85	-14.07
13	0.98	0.84	0.91	-14.57	13	14.85	19.26	17.05	25.85
14	0.26	0.25	0.26	-2.99	14	9.70	9.71	9.70	0.07
15	0.04	0.04	0.04	-15.77	15	1.77	1.70	1.73	-3.77
16	0.22	0.18	0.20	-22.64	16	1.62	1.70	1.66	4.71
17	0.46	0.62	0.54	29.34	17	2.33	2.77	2.55	17.08
18	0.51	0.58	0.54	11.64	18	2.72	2.59	2.66	-5.00
19	0.55	0.61	0.58	9.62	19	5.80	5.95	5.88	2.55
20	0.84	0.92	0.88	8.50	20	6.14	6.91	6.52	11.78
21	2.98	2.80	2.89	-6.18	21	9.56	9.16	9.36	-4.28
22	0.78	0.63	0.71	-22.45	22	7.73	12.87	10.30	49.86

The percent difference was calculated using the following equation: % difference = 100 × (repeat value −initial value) / mean value

Table S4. ESI monitoring mode and detailed MRM transition for each immunosuppressant.

Immunosuppressant	Monitoring mode	MRM transition		Collision energy	
		Quantification	Qualification	Quantification	Qualification
MPA	ESI negative	319.20 > 191.25	319.20 > 275.30	30 V	16 V
MPA-IS		323.20 > 191.25	323.20 > 279.25	23 V	18 V
MPAG		495.20 > 191.30	495.20 > 113.20	37 V	22 V
MPAG-IS		499.20 > 191.20	499.20 > 279.30	42 V	32 V
Tac	ESI positive	821.50 > 768.55	821.50 > 576.25	-22 V	-25 V
Tac-IS		826.50 > 773.60	826.50 > 581.50	-22 V	-22 V
Eve		975.60 > 908.55	975.60 > 926.55	-17 V	-12 V
Eve-IS		981.60 > 914.70	981.60 > 932.60	-17 V	-15 V
Sir		931.60 > 864.65	931.60 > 882.60	-17 V	-13 V
Sir-IS		935.60 > 864.65	935.60 > 882.60	-18 V	-13 V
CycA		1220.00 > 1202.95	1220.00 > 1185.00	-19 V	-36 V
CycA-IS		1232.00 > 1215.00	1232.00 > 1197.00	-19 V	-34 V

MPA and MPAG, including each IS, are monitored in ESI negative mode, while Tac, Eve, Sir, and CycA, including IS, are monitored in ESI positive mode.

Table S5. Concentration of each immunosuppressant for calibration points L1 to L6

Immunosuppressant	L1	L2	L3	L4	L5	L6
MPA (µg/mL)	0.1	0.5	5.0	10.0	25.0	50.0
MPAG (µg/mL)	1.0	5.0	25.0	50.0	125.0	250.0
Tac (ng/mL)	1.73	4.86	9.27	13.96	23.30	33.09
Eve (ng/mL)	2.04	4.88	9.20	14.77	24.84	36.88
Sir (ng/mL)	1.93	5.07	9.76	15.09	24.30	35.4
CycA (ng/mL)	26.07	106.5	453.7	1027.0	1574.0	1866.0

Concentrations of each calibration point from L1 to L6 (lowest to highest). Each concentration is indicated as in whole blood.
